# The Neuroglial Potassium Cycle during Neurotransmission: Role of Kir4.1 Channels

**DOI:** 10.1371/journal.pcbi.1004137

**Published:** 2015-03-31

**Authors:** Jérémie Sibille, Khanh Dao Duc, David Holcman, Nathalie Rouach

**Affiliations:** 1 Neuroglial Interactions in Cerebral Physiopathology, Center for Interdisciplinary Research in Biology, Collège de France, INSERM U1050, CNRS UMR 7241, Labex Memolife, PSL Research University, Paris, France; 2 Université Paris Diderot, Sorbonne Paris Cité, Paris, France; 3 IBENS, Ecole Normale Supérieure, INSERM U1024, CNRS UMR 8197, Paris, France; 4 Université Paris 6, Paris, France; University College London, UNITED KINGDOM

## Abstract

Neuronal excitability relies on inward sodium and outward potassium fluxes during action potentials. To prevent neuronal hyperexcitability, potassium ions have to be taken up quickly. However, the dynamics of the activity-dependent potassium fluxes and the molecular pathways underlying extracellular potassium homeostasis remain elusive. To decipher the specific and acute contribution of astroglial K_ir_4.1 channels in controlling potassium homeostasis and the moment to moment neurotransmission, we built a tri-compartment model accounting for potassium dynamics between neurons, astrocytes and the extracellular space. We here demonstrate that astroglial K_ir_4.1 channels are sufficient to account for the slow membrane depolarization of hippocampal astrocytes and crucially contribute to extracellular potassium clearance during basal and high activity. By quantifying the dynamics of potassium levels in neuron-glia-extracellular space compartments, we show that astrocytes buffer within 6 to 9 seconds more than 80% of the potassium released by neurons in response to basal, repetitive and tetanic stimulations. Astroglial K_ir_4.1 channels directly lead to recovery of basal extracellular potassium levels and neuronal excitability, especially during repetitive stimulation, thereby preventing the generation of epileptiform activity. Remarkably, we also show that K_ir_4.1 channels strongly regulate neuronal excitability for slow 3 to 10 Hz rhythmic activity resulting from probabilistic firing activity induced by sub-firing stimulation coupled to Brownian noise. Altogether, these data suggest that astroglial K_ir_4.1 channels are crucially involved in extracellular potassium homeostasis regulating theta rhythmic activity.

## Author Summary

Neural excitability relies on precise inward and outward ionic fluxes. In particular, potassium ions, released by neurons during activity, have to be taken up efficiently to prevent hyperexcitability. Astrocytes, the third element of the synapse, play a prominent role in extracellular potassium homeostasis. Thus unraveling the dynamics of the neuroglial potassium cycle during neurotransmission and the underlying molecular pathways is a key issue. Here, we have developed a tri-compartment model accounting for potassium dynamics between neurons, astrocytes and the extracellular space to quantify the specific and acute contribution of astroglial K_ir_4.1 channels to extracellular potassium levels and to the moment-to-moment neurotransmission. We demonstrate that astroglial K_ir_4.1 channels are sufficient to account for the slow membrane depolarization of astrocytes and crucially contribute to extracellular potassium clearance during basal and high activity. We also show that astrocytes buffer in less than 10 seconds more than 80% of the potassium released by neurons, leading to recovery of basal extracellular potassium levels and neuronal excitability. Remarkably, we found that K_ir_4.1 channels also prominently regulate slow 3 to 10 Hz rhythmic firing activity. Altogether, these data show that K_ir_4.1 channels acutely regulate extracellular potassium and neuronal excitability during specific patterns of activity.

## Introduction

Astrocytic processes enwrap more than half of CA1 hippocampal synapses to form tripartite synapses [1,2]. Perisynaptic astroglial processes are enriched in ionic channels, neurotransmitter receptors and transporters, enabling astrocytes to detect neuronal activity via calcium signaling [3] and ionic currents with various components, such as glutamate and GABA transporter [4–7] or potassium (K^+^) [8–10]. Thus astrocytes regulate neuronal activity through multiple mechanisms, involving signaling or homeostasis of extracellular space volume, glutamate, GABA or K^+^ levels [11]. Interestingly, membrane depolarization was the first activity-dependent signal identified in glial cells and was attributed to K^+^ entry across their membrane [10]. Such K^+^ entry was suggested to contribute to K^+^ spatial buffering, consisting in glial uptake of excess extracellular K^+^ ([K^+^]_o_), redistribution via gap-junction astroglial networks and subsequent release at sites of low [K^+^]_o_ [12].

Modeling studies have mostly investigated astroglial regulation of [K^+^]_o_ during pathological conditions to clarify its impact on aberrant neuronal activity. In particular astrocytes, by regulating [K^+^]_o_, have been shown to contribute to initiation and maintenance of epileptic seizures [13–15], as well as to the severity of ischemia following stroke, with a neuroprotective or neurotoxic role, depending on [K^+^]_o_ [16,17]. In addition, experimental data suggest that several K^+^ channels or transporters contribute to astroglial K^+^ clearance, such as inward rectifier 4.1 and two pore K^+^ channels (K_ir_4.1 and K_2P_, respectively) or Na/K ATPases [18,19]. Remarkably, recent work suggest that K_ir_4.1 channels play a prominent role in astroglial regulation of [K^+^]_o_ [20–23]. However, the mouse model used to draw these conclusions, i.e. conditional K_ir_4.1 knockout mice directed to glial cells (GFAP-Cre-K_ir_4.1fl/fl mice, K_ir_4.1^-/-^), exhibits several limitations: 1) K_ir_4.1 channels are not specifically deleted in astrocytes, but also in other glial cells such as oligodendrocytes or retinal Müller cells [22]; 2) astrocytes are severely depolarized [21,22]; 3) K_ir_4.1^-/-^ mice die prematurely (~3 weeks) and display ataxia, seizures, hindleg paralysis, visual placing deficiency, white matter vacuolization and growth retardation [22], highlighting that chronic deletion of K_ir_4.1 channels induces multiple brain alterations and possibly compensations. Thus, the specific and acute contribution of astroglial K_ir_4.1 channels to [K^+^]_o_ and to the moment to moment neurotransmission is still unclear. To decipher the acute role of astrocytes in controlling K^+^ homeostasis and neuronal activity, we built a tri-compartment model accounting for K^+^ dynamics between neurons, astrocytes and the extracellular space. We quantified K^+^ neuroglial interactions during basal and high activity, and found that K_ir_4.1 channels play a crucial role in K^+^ clearance and astroglial and neuronal membrane potential dynamics, especially during repetitive stimulations, and prominently regulate neuronal excitability for 3 to 10 Hz rhythmic activity.

## Results

### Modeling potassium dynamics between neuronal, glial and extracellular compartments

**Fig 1:**
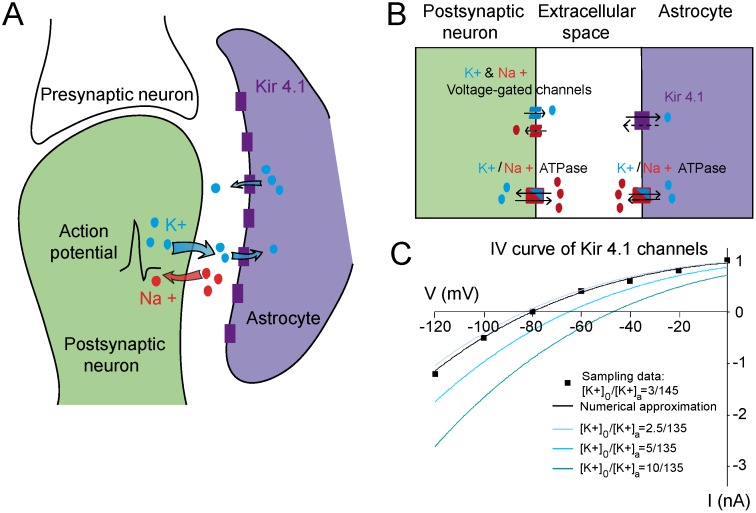
Tri-compartment model of the potassium cycle between the neuron, the extracellular space and the astrocyte. A, Schematic representation of the tri-compartment model: neuronal activity induces the release of K^+^ in the extracellular space, which is taken up by astrocytes. B, Reduction of the tri-compartment model to ionic fluxes exchanges between a generic postsynaptic neuron, astrocyte and extracellular space. The model includes channels and pumps carrying K^+^ and Na^+^ ions. C, Current-Voltage relationship (I-V curve) of K_ir_4.1 channels. We identify the free parameters in equation 22 by fitting the simulated IV curve (light blue) to experimental recordings performed in isolated astrocytes (sampling data, black rectangles) [65]. Using equation 22, we plot the I-V curve for different ratios of extracellular to intracellular astrocytic K^+^ concentrations (2.5/135 (light blue), 5/135 (blue) and 10/135 (dark blue). At resting membrane potential (-80 mV) and resting [K^+^]_o_ (2.5 mM), the K_ir_4.1 current is outward, but as illustrated here, it reverses by increasing [K^+^]_o_.

To model K^+^ ions dynamics during neuronal activity, we built a biophysical model that includes three compartments: the neuron, the astrocyte and the extracellular space (Fig. 1*A*). As performed in several studies [13,16,24], the neuron is approximated by a single compartment conductance-based neuron containing Na^+^ and K^+^ voltage-gated channels, enabling action potential discharge. The associated neuronal membrane potential is coupled with the dynamics of intracellular and extracellular Na^+^ and K^+^ levels via the dependence of the neuronal currents to the Nernst equation. The ion concentrations depend also on the activity of neuronal and astroglial Na/K ATPases, which maintain resting [K^+^]_i_ by balancing K^+^ and Na^+^ fluxes. Similarly, the astrocyte is approximated by a single compartment conductance-based astrocyte containing K_ir_4.1 channels, which are inward rectifier K^+^ channels strongly expressed in astrocytes that generate dynamic K^+^ currents [25]. In the model, neurons and astrocytes are separated by a homogenous extracellular space compartment. The model is based on balancing ionic fluxes between the three compartments (Fig. 1*B*). The model starts with the induction of a synaptic current (I_*app*_, see Materials and Methods). This current is the initial input of a classical Hodgkin-Huxley model, which describes the neuronal membrane potential dynamics (entry of Na^+^ and exit of K^+^). Released extracellular K^+^ is taken up by astrocytes through K_ir_4.1 channels and Na/K ATPases (Fig. 1*B* and Materials and Methods). Because K_ir_4.1 channels are strongly involved in K^+^ uptake [22], we fitted the I-V curve of K^+^ ions through K_ir_4.1 channels using equation 22 (see materials and methods) and predicted the I-V curve at various values of [K^+^]_o_ (Fig. 1*C*). We obtain that K^+^ fluxes through K_ir_4.1 channels vanish around astrocytic resting membrane potential (~-80 mV) and are outward during astrocytic depolarization for a fixed [K^+^]_o_ (2.5 mM, Fig. 1*C*). However, they become inward when [K^+^]_o_ increases (5–10 mM, Fig. 1*C*). Using this model, we shall investigate quantitatively the contribution of K_ir_4.1 channels to K^+^ uptake in relation to neuronal activity associated with different [K^+^]_o_.

### Astroglial membrane potential dynamics induced by stimulation

**Fig 2:**
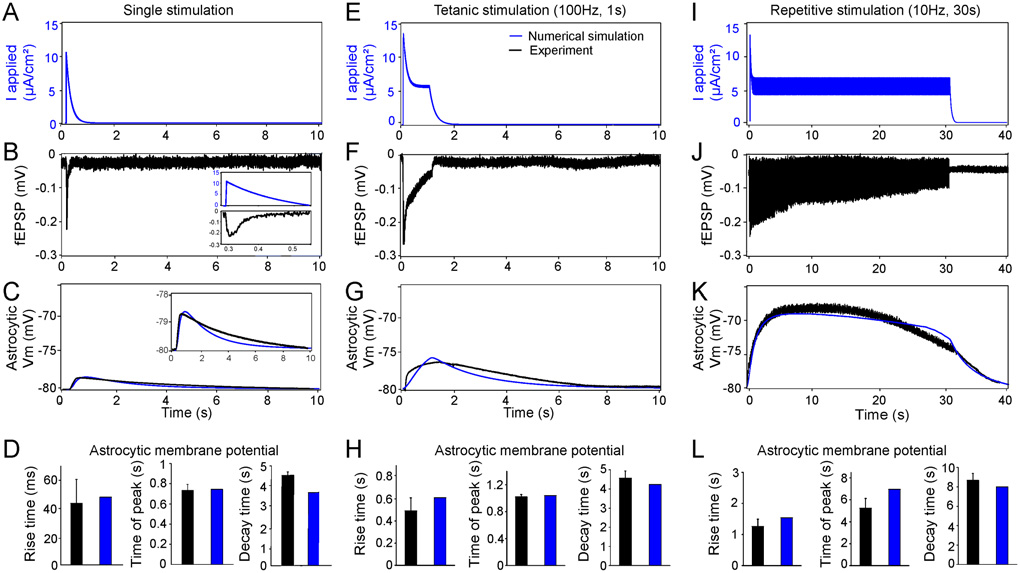
Dynamics of astroglial membrane potential induced by single, tetanic and repetitive stimulations. comparison of simulations and experiments ***A*,*E*,*I***, Numerical simulation of the applied current (I_*app*_, blue) induced by single (***A***), tetanic (100 Hz, 1 s) (***E***) and repetitive (10 Hz, 30 s) (***I***) stimulations generated by the depression-facilitation model with inputs ***f(t)* = *f_S_(t)*** (Equation 4), ***f(t)* = *f_TT_(t)*** (Equation 5) and ***f(t)* = *f_RS_(t)*** (Equation 6), respectively. *B*,*F*,*J*, Representative electrophysiological recordings of synaptic transmission (field excitatory postsynaptic potential, fEPSP, black) induced by single (***B***), tetanic (100 Hz, 1 s) (***F***) and repetitive (10 Hz, 30 s) (***J***) stimulations of Schaffer collaterals in acute hippocampal slices. Inset in panel B is a magnification of the simulated applied current (I_*app*_) illustrated in (***A***) and the corresponding experimental field excitatory postsynaptic potential (fEPSP) shown in (***B***). ***C*, *G*, *K***, Superimposition of astrocytic membrane potential dynamics obtained by electrophysiological recordings (black) and numerical simulations (blue) generated by equation 23 during single (***C***), tetanic (***G***) and repetitive stimulations (***K***). Inset in panel C is a magnification of the simulated and experimentally recorded astrocytic membrane potentials ***D*,*H*,*L***, Quantification of astrocytic membrane potential kinetics extracted from experimental data (black) and numerical simulations (blue). The rise and decay times are computed between 20% and 80% of the maximal peak amplitude response.

To validate our tri-compartment model, we compared simulation results with electrophysiological recordings. To account for the synaptic properties of CA1 pyramidal neurons, we generated a synaptic current (I_*app*_) using the depression-facilitation model (equation 1) (see Materials and Methods with input f(t) = δ(t)) (Fig. 2*A*,*E*,*I*). We first investigated responses to single stimulation. Using the Hodgkin-Huxley model, this synaptic current induces a firing activity (S1*A* Fig.), resulting in a ~ 0.9 mM increase of [K^+^]_o_ within 300 milliseconds, which slowly decayed back to baseline levels during 10 seconds (S1*B* Fig.). The extracellular K^+^ dynamics was associated in our model with a small astrocytic depolarization of ∆V = −1.35 mV (equations 22, 23, 25) (Fig. 2*C*). Using electrophysiological recordings of evoked field excitatory postsynaptic potential (fEPSP) by a single stimulation of Schaffer collaterals in acute hippocampal slices (Fig. 2*B*), we measured astroglial membrane potential depolarization and found that it reached ~ 1.3 mV (1.3 ± 0.2 mV, n = 6) (Fig. 2*C*), confirming the result of our simulation. After validating the responses of the tri-compartment model to basal stimulation, we investigated the impact of trains of stimulations on the dynamics of astroglial membrane potential. During tetanic stimulation (100 Hz for 1 second), variations in neuronal membrane potential described by the Hodgkin-Huxley equation show a bursting activity during ~ 1 second (S1*C* Fig.). This is associated with a depolarization of astrocytic membrane potential of ~ 5 mV, which lasts ~ 6 seconds (Fig. 2*G*,*H*) and an increase in [K^+^]_o_ that reaches a peak value of 4.4 mM (S1*D* Fig.).

For repetitive stimulations (10 Hz for 30 seconds), the neuron exhibited firing activity during the whole stimulation (S1*E* Fig.). This was associated with an astroglial depolarization of ~ 12 mV (Fig. 2*K*) and an increase in [K^+^]_o_ peaking at 6.9 mM after 17.5 seconds of stimulation (S1*F* Fig.). Although the stimulation lasted 30 seconds, the astrocytic depolarization started to decay after 17 seconds (Fig. 2*K*).

The kinetics of astroglial membrane potential dynamics obtained with the numerical simulations are comparable to the results obtained with electrophysiological recordings performed in individual astrocytes during single stimulation (rise time: 48.4 ms for numerical stimulation, 42 ± 19 ms n = 6 for experiments; time of peak: 740 ms for numerical simulation, 730 ± 60 ms n = 6 for experiments; decay time: 3.67 s for numerical simulation, 4.50 s ± 0.2 n = 6 for experiments, Fig. 2*D*), tetanic stimulation (rise time: 610 ms for numerical simulation, 491 ± 122 ms n = 5 for experiments; time of peak: 1.07 s for numerical simulation, 1.05 s ± 0.25 n = 5 for experiments; decay time: 4.18 s for numerical simulation, 4.55 s ± 0.45 n = 5 for experiments, Fig. 2*H*) and repetitive stimulation (rise time: 1.5 s for numerical simulation, 1.27 s ± 0.18 n = 5 for experiments; time of peak: 6.8 s for numerical simulation, 5.2 s ± 0.9 n = 5 for experiments; decay time: 7.95 s for numerical simulation, 8.3 s ± 0.4 n = 5 for experiments, Fig. 2*L*). These data show that the dynamics of astroglial membrane potential changes obtained from numerical simulations and from electrophysiological recordings are similar. Thus our model captures the key players sufficient to mimic the evoked astroglial membrane potential dynamics observed experimentally in different regimes of activity.

### Potassium redistribution in neuronal, astroglial and extracellular space compartments for different regimes of activity

**Fig 3:**
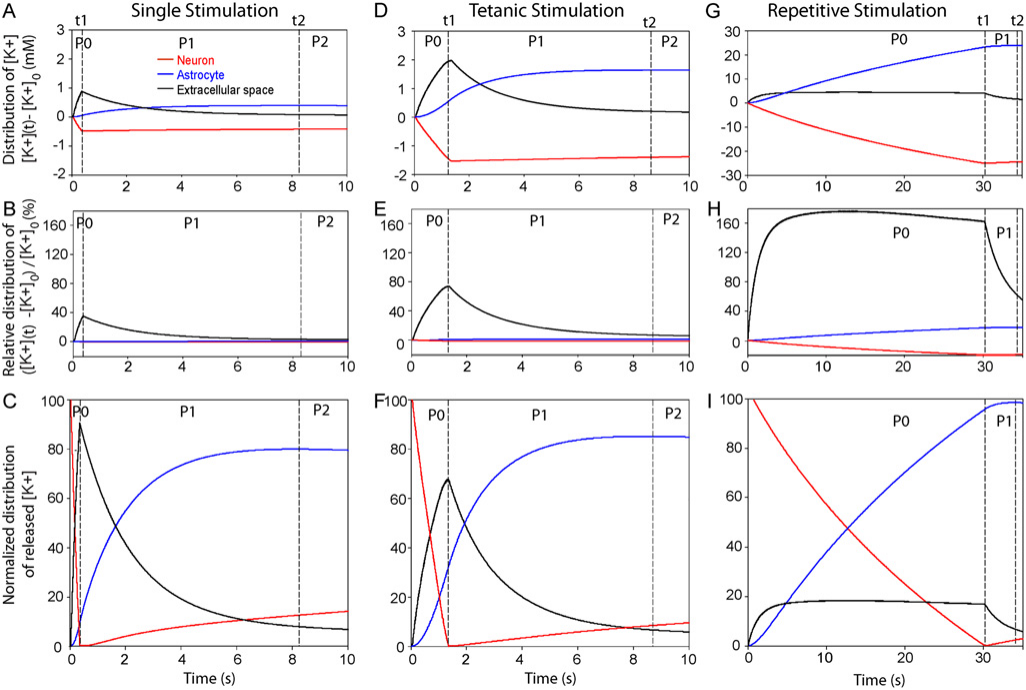
The potassium cycle between neuronal, astroglial and extracellular space compartments during basal and trains of stimulations. ***A-I***, K^+^ redistribution between neurons, extracellular space and astrocytes induced by single (***A-C***), tetanic (100 Hz, 1 s) (***D-F***) and repetitive (10 Hz, 30 s) (***G-I***) stimulations. For all regimes of activity, neuronal K^+^ (red) is released, increasing K^+^ in the extracellular space (black) during the stimulation initiated at time t = 0 (phase 0, t = 0 to t1), and is then cleared by the astrocyte (blue) (phase 1, t1 to t2). K^+^ levels are illustrated for the different regimes in each compartment (***A*,*D*,*G***) and are normalized to basal [K^+^]_o_ (***B*,*E*,*H***) or to the total amount of released K^+^ by neurons (***C*,*F*,*I***). Finally, the buffered K^+^ is slowly redistributed back to neurons, which ends the **K** ^+^ cycle (phase 2, t2 to end). t1 represents the time point where neuronal release of K^+^ stops, whereas t2 is the time point where astroglial K^+^ uptake peaks.

We investigated the dynamics of the K^+^ cycle between neurons, extracellular space and astrocytes induced by neuronal activity to decipher the time needed to restore basal extracellular and intra-neuronal K^+^ levels. We studied K^+^ redistribution induced by single, tetanic (100 Hz, 1 s) and repetitive (10 Hz, 30 s) stimulations, and found that the general behavior of K^+^ dynamics was divided into three phases (phases 0, 1 and 2; Fig. 3).

During phase 0 (t = 0 to t1), neuronal K^+^ is released in the extracellular space (peak [K^+^]_o_ during phase 0: 0.9 mM at 300 ms for single stimulation; 1.9 mM at 1.3 s for tetanic stimulation; 4.4 mM at 30 s for repetitive stimulation, Fig. 3*A*,*D*,*G*). Compared to basal K^+^ levels in each compartment (at t = 0), the relative transient evoked increase in K^+^ concentration is prominent only in the extracellular space (~+37% for single stimulation, +76% for tetanic stimulation and +168% for repetitive stimulation, Fig. 3*B*,*E*,*H*).

During phases 0 and 1, released K^+^ is then mostly buffered by astrocytes (~80 to 99% at the end of phase 1) during the different regimes of activity (time t2 (at the end of phase 1) for single stimulation: 8.2 s; tetanic stimulation: 8.7 s; repetitive stimulation: 34.2 s, Fig. 3*C*,*F*,*I*). The astroglial net K^+^ uptake increases with the activity-dependent [K^+^]_o_ transient rises (S2*A–C* Fig.) evoked by the different regimes (S2*D* Fig.). Neurons slowly re-uptake only ~5–10% of their released K^+^ at the end of phase 1 (Fig. 3*C*,*F*,*I*). Remarkably, although [K^+^]_o_ increases with the strength of stimulation (from 0.9 to 4.4 mM, Fig. 3*A*,*D*,*G* and S*2A–C* Fig.), the time needed for astrocytes to buffer the released K^+^ is not proportional to [K^+^]_o_ rises (Fig. 3*C*,*F*,*I*), as shown by the phase diagram illustrating astroglial K^+^ uptake as a dynamic function of activity-dependent changes in [K^+^]_o_ evoked by the different stimulations (S*2D* Fig.), but is to the square root of [K^+^]_o_ (equation 22). In addition, at the end of phase 1, [K^+^]_o_ is almost back to baseline levels, whereas intra-astroglial K^+^ levels reach their peak value (Fig. 3*C*,*F*,*I*).

Finally during phase 2 (t2 to end), astroglial buffered K^+^ is slowly redistributed back to neurons, which ends the K^+^ cycle. The long-lasting phase 2 is marked by an inversion of K^+^ fluxes in astrocytes, suggesting moderate K^+^ release by astrocytes over time. Indeed, K^+^ redistribution to neurons depends on K^+^ release through K_ir_4.1 channels, which is limited by the low outward rectification of these channels (Fig. 1*C*). Altogether, these data suggest a slow, but dynamic and efficient astroglial clearance capacity for the different regimes of activity.

### K_ir_4.1 channel contribution to neuronal firing and extracellular K^+^ levels

To study quantitatively the acute and selective role of astroglial K_ir_4.1 channels in neuroglial K^+^ dynamics, we inhibited the K_ir_4.1 current in our tri-compartment model. Because K_ir_4.1^-/-^ mice display altered synaptic plasticity compared to wild type mice [22,26], we recalibrated the synaptic current (I_*app*_) parameters τ_rec_ and τ_inact_ in equations 1,2 (see Table 1) for the facilitation-depression model to get an optimal fit to the recorded postsynaptic responses [26]. Another change in the model consisted in setting at zero both the K_ir_4.1 current and the leak term. In addition, to compensate for the loss of K^+^ fluxes through astroglial K_ir_4.1 channels, we added in equation 27 a constant K^+^ flux to maintain [K^+^]_o_ at an equilibrium value of 2.5 mM. Consequently, the astrocytic membrane potential displayed no change during stimulation, in agreement with electrophysiological recordings [21,22].

**Table 1:** Parameters.

	Parameters	Value
τ_*rec*_	Recovery time constant	300 ms (WT) / 500 ms (fitted KO) [55]
τ_*inac*_	Inactivation time constant	200 ms (WT) / 160 ms (fitted KO) [55]
*A*_*se*_	Absolute synaptic strength	7 (WT) / 10 (fitted KO) [55]
*U*_*se*_	Utilization of synaptic efficacy	0.8 (WT) / 0.8 (KO)
*g*_*Na*_	Neuronal sodium channel conductance	15 nS
*g*_*K*_	Neuronal potassium channel conductance	4 nS
*V*_*rest*_	Neuronal resting membrane potential	-60 mV [71]
*Na*	Avogadro Number	6.02 × 10^23^
*q*_*e*_	Net charge of single monovalent ion	1.62 × 10^–19^ C
*F*	Faraday Constant	9.64 × 10^–4^ C.mol^-1^
*R*	Gaz constant	8.314 J.mol^-1^.K^-1^
*T*	Temperature	308 K
*g*_*lN*_	Neuronal leak conductance	0.07 nS
*V*_*lN*_	Neuronal leak potential	-1.2793 mV
*C*_*N*_	Neuronal membrane capacitance	136 pF [53]
$G_{K_{ir}}$	Astrocytic single channel K_ir_ conductance	60 pS
*V*_*A*1_	K_ir_ current potential constant 1	- 14.83 mV extracted from [60]
*V*_*A*2_	K_ir_ current potential constant 2	34 mV extracted from [60]
*V*_*A*3_	K_ir_ current potential constant 3	19.23 mV extracted from [60]
*C*_*A*_	Astrocytic capacitance	15 pF [72]
*V*_*lA*_	Astrocytic leaking potential	-74 mV
*g*_*lA*_	Astrocytic leak conductance	0.1 nS
*i*_*maxA*_	Astrocytic Na/K pump rate	0.3 mM.ms^-1^
*i*_*maxN*_	Neuronal Na/K pump rate	0.9 μM.ms^-1^
$\frac{\;Vol_{o}}{\;Vol_{N}}$	Extracellular space volume/ neuronal volume	0.5 [73]
$\frac{\;Vol_{o}}{\;Vol_{A}}$	Extracellular space volume/astrocytic volume	0.5 [73]
*i*_*NalN*_	Neuronal sodium leak rate	-1.35.10^–4^ mM.ms^-1^
*i*_*NalA*_	Astrocytic sodium leak rate	-1.6.10^–3^ mM.ms^-1^

**Fig 4:**
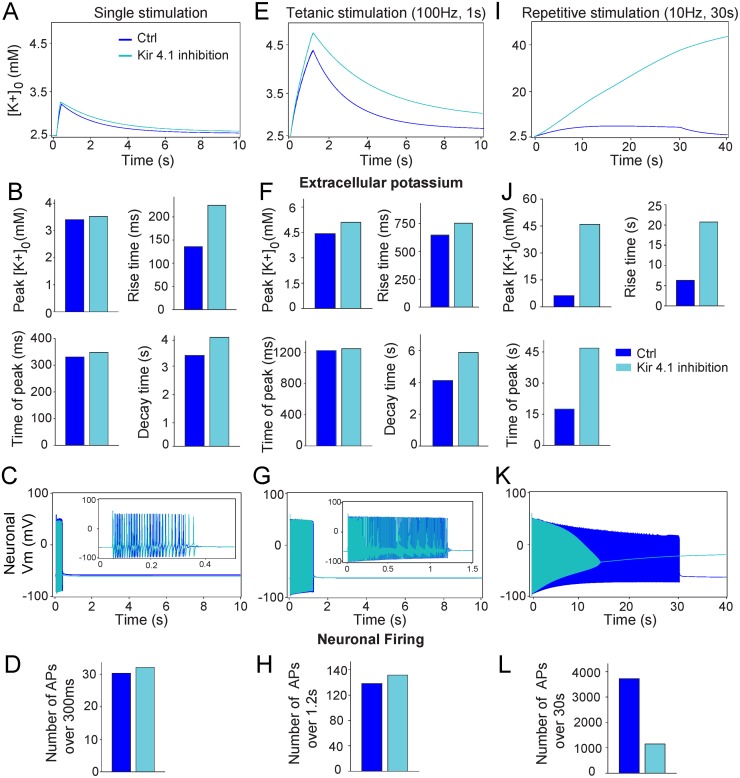
Acute contribution of astroglial K_ir_4.1 channels to the dynamics of neuronal firing and extracellular potassium levels. Comparison of simulated [K^+^]_o_ (***A*,*E I***) or neuronal firing (***C*,*G*,*K***) in control conditions (blue, Ctrl) and during inhibition of K_ir_4.1 channels (light blue) following single (***A-D***), tetanic (100 Hz, 1 s) ***(E-H)*** and repetitive (10 Hz, 30 s) (***I-L***) stimulations, respectively. Quantification of kinetics of extracellular K^+^ transients (***B*,*F*,*J***) and neuronal firing (***D*,*H*,*L***) evoked by single, tetanic and repetitive stimulations, respectively.

The numerical simulations show that inhibition of astroglial K_ir_4.1 channels leads to higher transient peak increase in [K^+^]_o_ during repetitive and tetanic stimulation compared to control conditions (Fig. 4*E*,*F*,*I*,*J*), while no difference is observed for single stimulation (Fig. 4*A*,*B*). In addition, for all regimes of activity, the rise and decay times of the [K^+^]_o_ were increased when K_ir_4.1 channels were inhibited (single stimulation, control: rise time 136 ms, decay time 3.4 s; K_ir_4.1 inhibition: rise time 232 ms, decay time 4.2 s; tetanic stimulation, control: rise time 638 ms, decay time 4 s; K_ir_4.1 inhibition: rise time 753 ms, decay time 6 s; repetitive stimulation, control: rise time 6.8 s; K_ir_4.1 inhibition: rise time 20.2 s, Fig. 4*B*,*F*,*J*).

Finally, K_ir_4.1 channel inhibition only slightly increased neuronal firing induced by single stimulation (Fig. 4*C*,*D*) and tetanic stimulation (Fig. 4*G*,*H*), while it had major effect on neuronal excitability during repetitive stimulation (Fig. 4*K*,*L*). Indeed, although firing frequency was only slightly increased during the first 8 seconds of repetitive stimulation when [K^+^]_o_ reached 10 mM (Fig. 4*I*), action potential amplitude and firing rate then progressively decreased due to neuronal depolarization (from-33 mV to-19 mV after 14 and 30 seconds of stimulation, respectively), suppressing neuronal firing after 14 seconds of stimulation (Fig. 4K). Altogether, these data show that astroglial K_ir_4.1 channels are prominently involved in K^+^ buffering during high level of activity, and thereby have a major impact on neuronal resting membrane potential controlling firing during trains of stimulations.

### Astrocytic K_ir_4.1 channels modulate firing probability induced by low frequency sub-firing stimulation in noisy neurons

**Fig 5:**
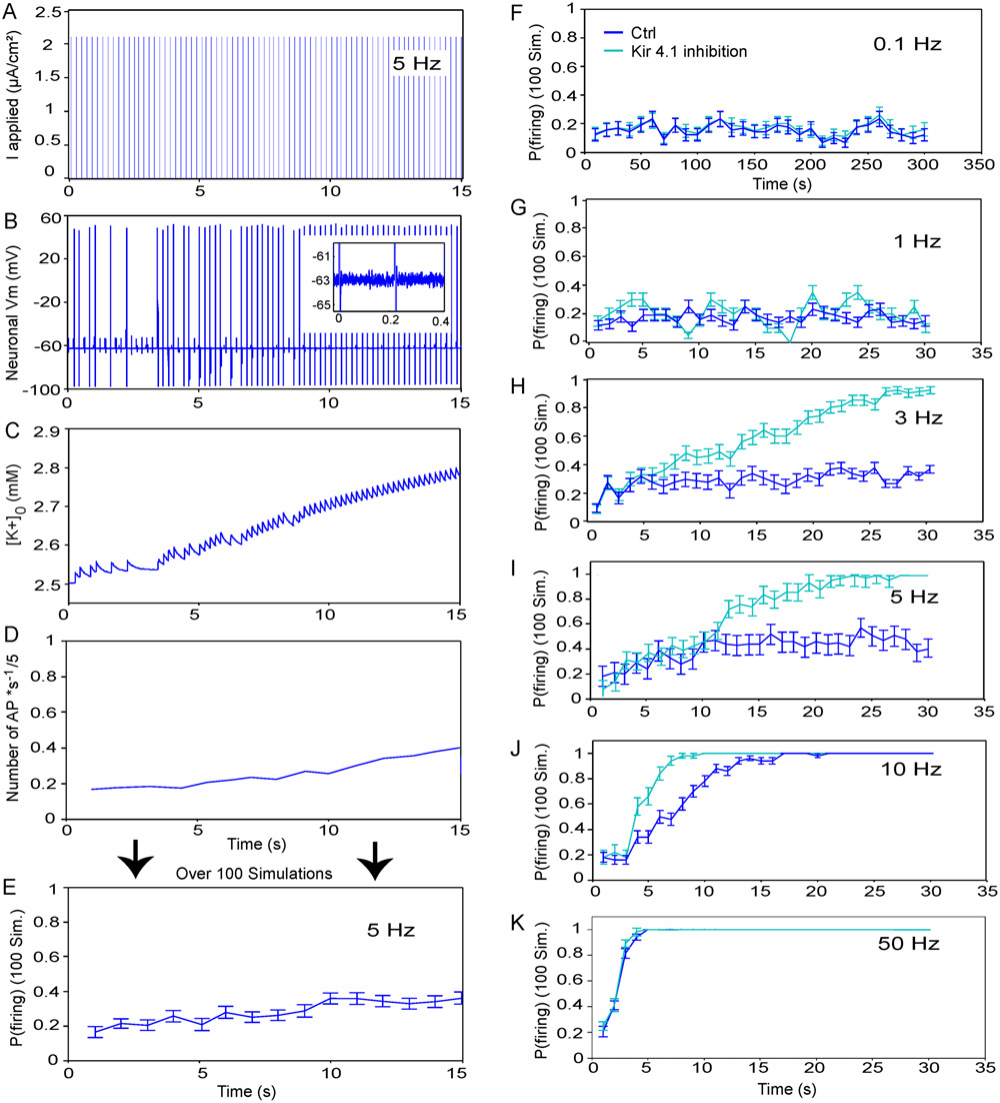
Involvement of K_ir_4.1 channels in firing probability induced by Brownian noise and sub-firing stimulation. ***A-E***, To induce probabilistic firing, a periodic sub-firing 5 Hz stimulation (5 ms squared stimulus) was set as the input of our tri-compartment model (***A***). Moreover, a Brownian source of amplitude **σ** = **0.68 *pA*** ^**2**^ **.*ms*** ^**-1**^ was added to induce a neuronal membrane potential noise of 1 mV amplitude (Inset in ***B***). Corresponding neuronal firing (***B***), [K^+^]_o_ (***C***) and estimated firing probability (number of action potentials (AP) per second/stimulation frequency) obtained by one simulation (***D***) are illustrated below for a 5 Hz stimulation during 15 seconds. ***E***, Quantification of the average firing probability computed over 100 numerical simulations for 5 Hz stimulation during 15 seconds in control condition. ***F-K***, Same quantification over time as in (***E***) in control (Ctrl, blue) and inhibited K_ir_4.1 channel (light blue) conditions illustrated for 0.1 Hz (***F***), 1 Hz (***G***), 3 Hz (***H***), 5 Hz (***I***), 10 Hz (***J***) and 50 Hz (***K***) stimulations.

To investigate the effect of astroglial K_ir_4.1 channels on endogenous physiological rhythmic activity, we generated probabilistic firing induced by sub-firing stimulation coupled to neuronal Brownian noise (Fig. 5*A*,*B*). To simulate the firing activity, we generated a sub-firing periodic stimulation (5 ms squared stimulus), which defines the applied synaptic intensity in our tripartite compartment model (Fig. 5*A*), and added a Brownian noise in the neuronal membrane potential (equation 21, Fig. 5*B*). Such stimulation induces an increase in [K^+^]_o_ (Fig. 5*C*), and thus firing over time (Fig. 5*D-E*). We found that astroglial K_ir_4.1 channels had no effect on the firing probability (computed over 100 simulations) for basal (0.1 Hz, Fig. 5*F*), low (1 Hz, Fig. 5*G*) and high (50 Hz, Fig. 5*K*) frequency stimulations. However, K_ir_4.1 channels directly regulate the firing probability for 3 and 5 Hz stimulations after 7 and 12 s of sub-firing stimulation, respectively (Fig. 5*H*,*I*). In contrast, K_ir_4.1 channels regulate only transiently the firing probability induced by 10 Hz stimulation (Fig. 5*J*). These data suggest a prominent and specific involvement of astroglial K_ir_4.1 channels in regulation of firing during theta rhythmic activity.

## Discussion

[K^+^]_o_ modulates neuronal membrane potential, excitability, release probability and synaptic efficacy [27–32]. To unravel the acute role of astrocytes in extracellular K^+^ homeostasis and neuronal activity, we used electrophysiological recordings with a tri-compartment model accounting for K^+^ dynamics between neurons, astrocytes and the extracellular space. We found that K_ir_4.1 channels play a key role in extracellular K^+^ clearance, astroglial and neuronal membrane potential dynamics, especially during trains of stimulation, and strongly regulate neuronal excitability for slow rhythmic activity (3–10 Hz).

### A novel tri-compartment model accounting for astroglial K_ir_4.1 channels and membrane potential dynamics in K^+^ regulation of neuronal activity

Several models have investigated extracellular K^+^ regulation of neuronal activity, including glial uptake mechanisms [13–17,24,33,34].

To study seizure discharges and spreading depression, a first tri-compartment model including the neurons, astrocytes and extracellular space was proposed [24], although the astrocytic membrane potential was not taken into account, and K^+^ accumulation in the interstitial volume was controlled by a first-order buffering scheme that simulated an effective glial K^+^ uptake system. With such model, after evoked firing, it took ~17 s for the neuronal membrane potential to return to resting values, via activation of Na/K ATPases. The model also predicted that elevated [K^+^]_o_ have a key role in the initiation and maintenance of epileptiform activity. In our study, we accounted for the astroglial modulation of K^+^ buffering capacity regulated by its membrane potential, and found that the biophysical properties of astrocytic membranes including K_ir_4.1 channels were sufficient to account for the long-lasting clearance of extracellular K^+^. Interestingly, we confirm that alteration in K^+^ clearance leading to an extracellular K^+^ accumulation induces epileptiform activity, and show specifically that K_ir_4.1 channel acute inhibition leads to such pathological bursting activity during repetitive stimulation.

A similar tri-compartment model has been simplified as a one-dimensional two-layer network model to study how neuronal networks can switch to a persistent state of activity, as well as the stability of the persistent state to perturbations [13]. In this model, Na^+^ and K^+^ affect neuronal excitability, seizure frequency, and stability of activity persistent states. In particular, the quantitative contribution of intrinsic neuronal currents, Na/K ATPases, glia, and extracellular Na^+^ and K^+^ diffusion to slow and large-amplitude oscillations in extracellular and neuronal Na^+^ and K^+^ levels was revealed. In the model, the estimated [K^+^]_o_ during epileptiform activity are comparable to the ones observed experimentally [35,36]. Although this model does not account for astroglial K_ir_4.1 channels, it shows that a local persistent network activity not only needs balanced excitation and inhibition, but also glial regulation of [K^+^]_o_ [15].

Finally, a model accounting for the extracellular space and astroglial compartments has quantified the involvement of several astroglial ionic channels and transporters (Na/K ATPase, NKCC1, NBC, Na^+^, K^+^, and aquaporin channels) in the regulation of firing activity [34].

To account for K^+^ dynamics between neurons, astrocytes and the extracellular space, we built for the first time a tri-compartment model, where we included neuronal voltage-gated channels, Na/K pumps and astrocytic K_ir_4.1 channels according to their biophysical properties, as well as membrane potential of astrocytes. Because functional expression of voltage-gated calcium channels on hippocampal mature astrocytes *in situ* in physiological conditions and its impact on astrocytic functions is still a matter of debate [37], such channels were not included in our model. However, many other astroglial K^+^ channels (such as two pore domain K^+^ channels (K_2P_) (TWIK-1, TREK-1, TREK-2 and TASK-1), inward rectifier K^+^ channels (Kir2.1, 2.2, 2.3, 3.1, 6.1, 6.2), delayed rectifier K^+^ channels (Kv1.1, 1.2, 1.5, 1.6), rapidly inactivating A-type K^+^ channels (Kv1.4), calcium-dependent K^+^ channels (K_Ca_3.1)), but also other channels, transporters or exchangers (such as Cx hemichannels, Na^+^/K^+^/Cl^-^ co-transporter (NKCC1) K^+^/Cl^-^ exchanger, glutamate transporters) [16,38,39] could also play a role in the regulation of activity-dependent changes in [K^+^]_i_ or [K^+^]_o_. Functional evidence of the contribution of these channels, transporters or exchangers in astroglial K^+^ clearance is actually scarce, although K_2P_ channels have been suggested to participate in astroglial K^+^ buffering [40], while NKCC1 were recently shown in hippocampal slices not to be involved in activity-dependent K^+^ clearance [41]. Similarly, adding slower timescale K^+^ dependent conductances in the neuron model could modulate the slow redistribution of K^+^ to neurons, and thus the duration of the neuroglial potassium cycle, and is of interest to implement in future development of the model. In our study, the aim was to simplify the system to capture in the model the minimal set of astroglial channels and pumps accounting for our experimental data related to activity-dependent changes in astroglial membrane potential. In addition our tri-compartment model, as most existing models, did not account for the complex multiscale geometry of astrocytes and neurons. Incorporating in our current model additional astroglial and neuronal channels, as well as complex cell geometry is of particular interest to identify modulatory effects of other specific channels and of microdomain geometry on the neuroglial potassium cycle.

In accordance with previous studies, where K_ir_4.1 channels were chronically deleted genetically in glial cells [20,21,23], we found that acute inhibition of K_ir_4.1 channels leads to altered regulation of extracellular K^+^ excess and affects the kinetics of [K^+^]_o_ (Fig. 4*I*,*J*). However, in contrast to these studies, we found that K_ir_4.1 channel inhibition also alters significantly [K^+^]_o_ peak amplitudes during repetitive stimulation, suggesting that K_ir_4.1^-/-^ mice may display some compensatory mechanisms attempting to maintain extracellular K^+^ homeostasis. In addition, our model reveals that specific and acute inhibition of K_ir_4.1 channels slows down, but does not abolish, astroglial uptake of excess K^+^ during single, tetanic and repetitive stimulations, confirming that astroglial Na/K ATPases, included in our model, also contribute to K^+^ clearance [41].

### The long-lasting astrocytic potassium uptake is due in part to the slow K_ir_4.1 conductance dynamics

Contrary to action potentials, characterized by a very fast dynamics in the order of a few milliseconds, astroglial K^+^ buffering lasts tens of seconds. As shown in the present study, most of extracellular K^+^ released by neurons is first cleared by astrocytes through K_ir_4.1 channels. To determine the factors controlling the slow timescale of astroglial K^+^ clearance, we focused on K_ir_4.1 channels. Because the astroglial leak conductance (equation 23) is six times smaller than the K_ir_4.1 channel conductance, we neglected it.

The dynamics of astrocytic membrane potential *V*_*A*_ is described by equation 23, where the membrane capacitance is *C*_*A*_ ≈ 15 *pF* and the maximal K_ir_4.1 channel conductivity is$G_{K_{ir}}\approx 60pS$. In that case, using equation 23, the time constant of K_ir_4.1 channel-mediated return to equilibrium of astroglial membrane potential *τ*_*A*_ is defined as

$$\tau_{A}\approx C_{A}(1+\text{exp}(\frac{V_{A}-V_{KA-V_{2A}}}{V_{3A}}))/\left(G_{K_{ir}}\sqrt{K_{0}}\right)$$

We obtain the following approximation *τ*_*A*_ ≈ 0.6*s* using equation 23 and the parameters of table 1. This time constant is consistent with the fitted exponential decay time obtained in our simulations and experiments for a single stimulation where we obtained *τ* ≈ 0.7*s*. However, simulations for stronger stimulations indicate an increase of *τ* to approximatively 4 seconds (tetanic stimulation) and 9 seconds (repetitive stimulation). This increase in clearance duration is due to the dependence of the K_ir_4.1 current to [K^+^]_o_, as illustrated by the IV relation (Fig. 1*C*) and described in equation 22. The Nernst potential *V*_*KA*_ increases for strong stimulations (tetanic and repetitive), which slow down the kinetics of astrocytic membrane potential *V*_*A*_ through the term $1+\exp\left(\frac{V_{A}{}_{-}V_{KA-}V_{2A}}{V_{3A}}\right)$ in equation 22. We conclude that the slow time scale of K^+^ clearance is in part due to the availability of K_ir_4.1 channels at low and high [K^+^]_o_. This clearance timescale is much longer than the glutamate clearance rate of *τ*_*glu*_ ≈ 15 *ms* that we previously reported [42]. Moreover, the redistribution of K^+^ released by neurons during the different regimes of activity shows that the higher the activity, the lower the proportion of released K^+^ remains transiently in the extracellular space. This suggests that K_ir_4.1 channels have a strong uptake capacity, especially for high regimes of activity ([K^+^]_o_ up to 5–6 mM).

### Impact of K_ir_4.1-mediated potassium buffering on neuronal activity in physiology and pathology

Remarkably, our model reveals that astroglial K_ir_4.1 channels strongly regulate neuronal firing induced by high stimulation regime such as repetitive stimulation. K_ir_4.1channels are crucially involved in regulation of [K^+^]_o_ during this regime of activity, most likely because such stimulation triggered long-lasting neuronal release of K^+^ (20 mM over 30 seconds, Fig. 3*G*) resulting in a sustained, but moderate increase in [K^+^]_o_ (>6 mM for ~22 s, Fig. 4*I*,*J*), compared to the neuronal release. These data suggest that during repetitive stimulation, astrocytes can buffer up to ~14 mM of [K^+^]_o_ and thereby preserve neuronal firing. However, astroglial K_ir_4.1 channels slightly impact neuronal firing induced by single and tetanic stimulations, probably because these stimulations only triggered transient neuronal K^+^ release (0.9 mM over 300 ms (Fig. 3*A*) and 1.9 mM over 1.3 s (Fig. 3*D*), respectively), resulting in a short and small increase in [K^+^]_o_ (>2.7 mM for ~450 ms for single stimulation (Fig. 4*A*,*B*), and >3.5 mM for 1.5 s for tetanic stimulation (Fig. 4*E*,*F*)). Nevertheless, we show a prominent and specific involvement of astroglial K_ir_4.1 channels in probabilistic firing activity induced by 3 to 10 Hz sub-firing stimulations (Fig. 5), suggesting a key role of these channels in sustained theta rhythmic activity. Interestingly, these data imply that K_ir_4.1 channels can contribute to fine tuning of neuronal spiking involving low, but long-lasting, increase in [K^+^]_o_. Thus besides gliotransmission, regulation of [K^+^]_o_ by K_ir_4.1 channel provides astrocytes with an alternative active and efficient mechanism to regulate neuronal activity. Several studies have reported decreased K_ir_4.1 protein levels and K_ir_ functional currents in sclerotic hippocampus from epileptic patients [43–46]. Whether these changes are the cause or the consequence of epilepsy is still an open question. However, K_ir_4.1^-/-^ mice display an epileptic phenotype [22,47] and missense mutations in KCNJ10, the gene encoding K_ir_4.1, have been associated with epilepsy in humans [48,49]. These data thus suggest that impairment in K_ir_4.1 function leading to alterations in [K^+^]_o_ dynamics, as shown in our study, may cause epilepsy. Remarkably, dysfunction of [K^+^]_o_ regulation by K_ir_4.1 channels is likely involved in other pathologies, since it contributed to neuronal dysfunction in a mouse model of Huntington’s disease [50] and the presence of antibodies against K_ir_4.1 channels in glial cells was recently found in almost 50% of multiple sclerosis patients [51]. Thus astroglial K_ir_4.1 channels may well represent an alternative therapeutic target for several diseases.

## Materials and Methods

### Ethics statement

Experiments were carried out according to the guidelines of European Community Council Directives of January 1^st^ 2013 (2010/63/EU) and our local animal committee (Center for Interdisciplinary Research in Biology in Collège de France). All efforts were made to minimize the number of used animals and their suffering. Experiments were performed on the hippocampus of wild type mice (C57BL6). For all analyses, mice of both genders and littermates were used (PN19–PN25).

### Electrophysiological recordings

Acute transverse hippocampal slices (400 μm) were prepared as previously described [42,52–54] from 19–25 days-old wild type mice. Slices were kept at room temperature (21–23°C) in a chamber filled with an artificial cerebrospinal fluid (ACSF) composed of (in mM): 119 NaCl, 2.5 KCl, 2.5 CaCl_2_, 1.3 MgSO_4_, 1 NaH_2_PO_4_, 26.2 NaHCO_3_ and 11 glucose, saturated with 95% O_2_ and 5% CO_2_, prior to recording. Acute slices were placed in a recording chamber mounted on a microscope including infra-red differential interference (IR-DIC) equipment, and were bathed in ACSF perfused at 1.5 ml/min. ACSF contained picrotoxin (100 μM), and connections between CA1 and CA3 regions were cut to avoid epileptic-like activity propagation. Extracellular field and whole-cell patch-clamp recordings were obtained using glass pipettes made of borosilicate. Astroglial and postsynaptic responses were evoked by Schaffer collateral stimulation (0.05Hz) in the CA1 *stratum radiatum* region with glass pipettes filled with ACSF (300–700 kΩ). Astrocytes from *stratum radiatum* were recognized by their small soma size (5–10 μm), very low membrane resistance and hyperpolarized resting membrane potentials (≈- 80 mV), passive properties of their membrane (linear I-V), absence of action potentials, and large coupling through gap junctions. Field excitatory postsynaptic potentials (fEPSPs) were obtained in 400 μm slices using pipettes (4–6 MΩ) located in the *stratum radiatum* region. Stimulus artifacts were suppressed in representative traces. Whole-cell recordings were obtained from CA1 astrocytes, using 4–6 MΩ glass pipettes containing (in mM): 105 K-Gluconate, 30 KCl, 10 HEPES, 10 Phosphocreatine, 4 ATP-Mg, 0.3 GTP-Tris, 0.3 EGTA (pH 7.4, 280 mOsm). Prolonged repetitive stimulation was performed for 30 s at 10 Hz. Post-tetanic potentiation was evoked by stimulation at 100 Hz for 1 s in the presence of 10 μM CPP ((Rs)-3-(2-Carboxypiperazin-4-yl-)propyl-1-phosphonic acid). Recordings were performed with Axopatch-1D amplifiers (Molecular Devices, USA), at 10 kHz, filtered at 2 kHz, and analyzed using Clampex (Molecular Devices, USA), and Matlab (MathWorks, USA) softwares. The data represent mean ± SEM. Picrotoxin was from Sigma and CPP from Tocris.

### Modeling potassium dynamics in the tripartite neuron-astrocyte-extracellular compartment

We present here the biophysical model we have built to describe K^+^ dynamics during neuronal activity and specifically the role of astroglial K_ir_4.1 channels. After Schaffer collateral stimulation, excitatory synapses release glutamate molecules that activate postsynaptic neurons. We modeled this step by classical facilitation/depression model [55]. The resulting postsynaptic activity triggers ionic release in the extracellular space and a change in the astrocytic membrane potential through ion uptake. We used the average neuronal potential and mass conservation equations for ionic concentrations to model changes in astrocytes. We have built a tri-compartment model, which accounts for: 1) the neuron, 2) the astrocyte and 3) the extracellular space. We included voltage gated channels, Na/K pumps and astrocytic K_ir_4.1 channels.

### Facilitation-depression model

To account for the stimulation of Schaffer collaterals that induce a postsynaptic response in the CA1 *stratum radiatum* region, we used a facilitation-depression model [55–57].

$$\frac{dr}{dt}=\frac{i}{\tau_{rec}}-U_{se}rf\left(t\right)$$ (1)

$$\frac{de}{dt}=-\frac{e}{\tau_{inac}}+U_{se}rf\left(t\right)$$ (2)

i=1−r−e (3)

where *f* is the input function. For a single stimulation generated at time *t*_*stim*_, *f*(t) = δ(t-*t*_*stim*_).

A stimulation instantaneously activates a fraction *U*_*se*_ of synaptic resources *r*, which then inactivates with a time constant *τ*_*inac*_ and recovers with a time constant *τ*_*rec*_ In the simulations, at time *t* = *t*_*stim*_, *r* and *e* respectively decreases and increases by the value *U*_*se*_ *r*. The synaptic current *I*_*app*_ is proportional to the fraction of synaptic resources in the effective state *e* and is given by *I*_*app =*_ *A*_*se*_ *e* (the parameter *A*_*se*_ is defined in table 1). We used the following definitions for the input function *f*:

$$f(t)\{\begin{array}{c} \;\;\;\;f_{s}\left(t\right)=\delta \left(t\right)\;\;\;\;\;\;\;\;for\;single\;stimulation\;\;\;\;\;\;\;\;\;\;\;\;\;\;\;\;\;\;\;\;\;\;\;\;\;\left(4\right)\\ \;f_{TT}\left(t\right)=\overset{100}{\underset{k=1}{\sum }}\delta \left(t+0.01k\right)\;for\;tetanic\;stimulation\;\left(100Hz\;for\;1\;\text{second}\right)\;\;\;\;\;\;\;\;\left(5\right)\\ \;\;f_{RS}\left(t\right)=\overset{300}{\underset{k=1}{\sum }}\delta \left(t+0.1k\right)\;\;for\;repetitive\;stimulation\;\left(10Hz\;for\;30\;seconds\right)\;\;\;\left(6\right) \end{array}\}$$

### Modeling neuronal activity

The dynamics of the neuronal membrane potential, *V*_*N*_, follows the classic Hodgkin Huxley (HH) equations [58].

$$I_{Na}=g_{Na}m^{3}h\left(V_{N}-V_{rest}+V_{NaN}\right)$$ (7)

$$I_{K}=g_{K}n^{4}\left(V_{N}-V_{rest}+V_{KN}\right)$$ (8)

$$\frac{dn}{dt}=\alpha_{n}\left(1-n\right)-\beta_{n}n$$ (9)

$$\frac{dm}{dt}=\alpha_{m}\left(1-m\right)-\beta_{m}m$$ (10)

$$\frac{dh}{dt}=\alpha_{h}\left(1-h\right)-\beta_{h}h$$ (11)

with rate equations

$$\alpha_{n}\left(V_{N}\right)=\frac{0.01\left(V_{N}+10\right)}{\exp\left(0.1\left(V_{N}+10\right)\right)-1}$$ (12)

$$\beta_{n}\left(V_{N}\right)=0.125\exp\left(V_{N}/80\right)$$ (13)

$$\alpha_{m}\left(V_{N}\right)=\frac{0.1\left(V_{N}+25\right)}{\exp\left(0.1\left(V_{N}+25\right)\right)-1}$$ (14)

$$\beta_{m}\left(V_{N}\right)=4\exp\left(V_{N}/18\right)$$ (15)

$$\alpha_{h}\left(V_{N}\right)=0.07\exp\left(V_{N}/20\right)$$ (16)

$$\beta_{h}\left(V_{N}\right)=\frac{1}{\exp\left(0.1\left(V_{N}+30\right)\right)+1}$$ (17)

*V*_*rest*_ is the resting membrane potential and *V*_*KN*_ and *V*_*NaN*_ are respectively the K^+^ and Na^+^ equilibrium potentials and are given by the Nernst equations

$$V_{NaN}=\frac{RT}{F}\ln\left(\frac{Na_{0}}{Na_{N}}\right)$$ (18)

$$V_{KN}=\frac{RT}{F}\ln\left(\frac{K_{0}}{K_{N}}\right)$$ (19)

where *N*a_0_ and *N*a_*N*_ are respectively the extracellular and neuronal sodium concentrations, and *K*_0_ and *K*_*N*_ are respectively the extracellular and neuronal K^+^ concentrations that may vary as we shall describe below. We complete the description of all the neuronal currents with a leak current

$$I_{lN}=g_{lN}\left(V_{N}-V_{lN}\right)$$ (20)

which stabilizes the membrane potential at its resting value. Finally, the neuronal membrane potential satisfies the equation

$$C_{N}\frac{dV_{N}}{dt}=-\left(I_{Na}+I_{K}+I_{lN}+I_{app}\right)$$ (21)

where *I*_*app*_ is the synaptic current derived from equation 1.

### Modeling astrocytic potassium uptake by K_ir_4.1 channels

To account for the K^+^ dynamics in astrocytes, we modeled the K_ir_4.1 channel according to its biophysical properties [59] and I-V curve [60]. The total astroglial current *I*_*Kir*_ depends on the membrane potential, the extracellular (*K*_0_) and the astrocytic (*K*_*A*_) K^+^ concentrations, and is approximated by

$$I_{Kir}=G_{K_{ir}}\left(V_{A}-V_{KA}-V_{A1}\right)\left(\frac{\sqrt{K_{0}}}{1+\exp\left(\frac{V_{A}-V_{KA}-V_{A2}}{V_{A3}}\right)}\right)$$ (22)

where V_KA_ is the Nernst astrocyte K^+^ potential, V_A_, the astrocyte membrane potential, K_0_ is the extracellular K^+^ concentration and V_A1_ (an equilibrium parameter, which sets K_ir_ current to 0 at-80 mV), V_A2_ and V_A3_ are constant parameters calibrated by the I-V curve (Fig. 1C, [60]), as detailed below. The second term of equation 22 describes the dependence of I_Kir_ to the square root of K_0_ [60–64] and to the steady state open/close partition function of Kir4.1 channels according to the Boltzmann distribution [59], which includes dynamic variations of potassium Nernst potential during neuronal activity.

Adding a leak current I_lA_ = g_lA_(V_A_—V_lA_), which stabilizes the astrocyte membrane potential at—80 mV, the astrocyte membrane potential *V*_*A*_ satisfies the equation

$$C_{A}\frac{dV_{A}}{dt}=-\left(I_{Kir}+I_{lA}\right)$$ (23)

where *I*_*Kir*_ is defined by relation 22.

We fitted the K_ir_4.1 channel I-V curve (equation 22) using the experimental recordings for the K_ir_4.1 channel (3 mM [K^+^] (Fig. 4 in [60,65]). We first obtained that *V*_*A1*_ = (*V*_*restA*_ − 26*ln*(3/145)) = −14.83 *mV* where *V*_*restA*_ = −80*mV* (potential for which the current is zero). We then used the Matlab fitting procedure for a single exponential with formula 22 changed to $\frac{(V-V_{A1}-26ln\left(3/145\right))\sqrt{3}}{I}$ with (*V* from-100 to 20 mV) to get that *V*_*A2*_ = 34 *mV* and *V*_*A3*_ = 19.23 *mV* (table 1). Varying [K^+^]_o_ by 0.5 mM did not affect significantly the K_ir_4.1 channel I-V curve, confirming its robustness.

### Na/K pump ionic flux for astrocytes and neurons

The K^+^ resting concentrations in neurons and astrocytes are maintained by Na/K pumps that balance the outward K^+^ and inward Na^+^ fluxes. The associated pump currents *i*_*pump*,*k*_ (index *k* = *N* for the neuron, *k* = *A* for the astrocyte) depend on the extracellular K^+^ *K*_0_ and intracellular Na^+^ concentrations (Na_N_ for the neuron and Na_A_ for the astrocyte) and follow the same equation as [66],

$$i_{pump,k}=i_{maxk}{\left(1+\frac{7.3}{K_{0}}\right)}^{-2}{\left(1+10Na_{k}\right)}^{-3}\;for\;k=N,A$$ (24)

where *i*_*maxk*_ is a constant (table 1).

### Balance of ionic fluxes

We converted the different electrogenic neuronal and astrocytic channel currents into ionic fluxes [13]. A current *I* across a membrane induces a flow of charge *i* equals to *δQ* = *I* per unit of time. The corresponding change in extracellular concentration is given by *I*/(*qN*_*A*_ *Vol*_0_), where *q* = 1.6 * 10^–19^C is the charge of an electron, *N*_*A*_ the Avogadro number and *Vol*_*N*_, *Vol*_*A*_ and*Vol*_*0*_ are the neuronal, astrocytic and extracellular volume respectively. To model the ionic concentration dynamics, we converted the currents *I*_*Na*_, *I*_*K*_ and *I*_*Kir*_ to the corresponding ionic fluxes *i*_*N*a_, *i*_*K*_ and *i*_*Kir*_ We describe in the following paragraphs the equations for the ionic concentrations in the three compartments (neuron, extracellular space and astrocyte).

### Potassium fluxes

To determine the system of equations for the K^+^ fluxes, we use the mass conservation law for the extracellular *K*_0_, the neuronal *K*_N_ and the astrocytic *K*_*A*_ K^+^ concentrations. The extracellular K^+^ *K*_0_ increases with the neuronal current *I*_*K*_ (see equation 8), which is here converted to *i*_*K*_ (ion flux), but it is also uptaken back into neurons with a flux 2 *i*_*pumpN*_ (the factor 2 is described in [67] and into astrocytes as the sum of the two fluxes 2 *i*_*pumpA*_ plus *i*_*Kir*_. Similarly, we obtain the equations for the neuronal and astrocytic K^+^ to balance the various fluxes. Finally, we get

$$\frac{dK_{0}}{dt}=i_{K}-2i_{pumpN}-2i_{pumpA}+i_{Kir}$$ (25)

$$\frac{dK_{N}}{dt}=\left(-i_{K}+2i_{pumpN}\right)\frac{Vol_{o}}{Vol_{N}}$$ (26)

$$\frac{dK_{A}}{dt}=\left(-i_{Kir}+2i_{pumpA}\right)\frac{Vol_{o}}{Vol_{A}}$$ (27)

To study quantitatively the acute and selective role of astroglial K_ir_4.1 channels in neuroglial K^+^ dynamics, we inhibited the K_ir_4.1 current in our tri-compartment model. We thus set at zero both the K_ir_4.1 current and the leak term. To compensate for the loss of K^+^ fluxes through astroglial K_ir_4.1 channels, we added in equation 27 a constant K^+^ flux to maintain [K^+^]_o_ at an equilibrium value of 2.5 mM. This constant K^+^ flux in astrocytes could be mediated by various channels or transporters such as two pore domain potassium channels (K2P such as TWIK-1, TREK-1, TREK-2 and TASK-1), delayed rectifier potassium channels (Kv1.1, 1.2, 1.5 and 1.6), rapidly inactivating A-type potassium channels (Kv1.4), glutamate transporters or connexin43 hemichannels. However, since TASK-1 [68] and Cx43 hemichannels [69] are thought to be active in basal conditions, they are more likely to mediate such flux.

### Sodium fluxes

Similarly to the K^+^ dynamics, the equations for the Na^+^ fluxes are derived using the balance between the neuronal, astrocytic and extracellular concentrations. However, the main differences are that the pump exchanges 2 K^+^ for 3 Na^+^ ions, leading to the coefficient 3 in front of the pump term. In addition, to stabilize the sodium concentrations, we added two constant leak terms *i*_*NalA*_ and *i*_*NalN*_ (values given in table 1), as classically used [24],

$$\frac{dNa_{0}}{dt}=i_{Na}+i_{NalN}+3i_{pumpN}+3i_{pumpA}+i_{NalA}$$ (28)

$$\frac{dNa_{N}}{dt}=\left(-i_{Na}-3i_{pumpN}-i_{NalN}\right)\frac{Vol_{o}}{Vol_{N}}$$ (29)

$$\frac{dNa_{A}}{dt}=\left(-i_{NalA}-3i_{pumpA}\right)\frac{Vol_{o}}{Vol_{A}}$$ (30)

### Numerical implementations and fitting procedures

**Numerical simulations**. Simulations, numerical integrations and fitting computations were performed in Matlab. We used Runge Kunta fourth order method for the simulations, which were numerically stable. We used a time step of ∆*t* = 0.1 *ms* (simulations were repeated with smaller time step to check whether numerical accuracy was affecting results). The leak currents parameters were adjusted to stabilize the model at the resting membrane potentials (- 70 mV and - 80 mV for neurons and astrocytes respectively) and resting concentrations (neuronal [K^+^] and [Na^+^]: 135 mM and 12 mM, respectively; extracellular [K^+^] and [Na^+^]: 2.5 mM and 116 mM, respectively; astrocytic [K^+^] and [Na^+^]: 135 mM and 12 mM, respectively). The parameters for the Hodgkin Huxley equations were also adjusted to these concentrations.

**Approximation of time constants**. Time constants τ of simulated extracellular K^+^ transients were fitted to curves using a single exponential $\left(e^{-\frac{t}{\tau }}\right)$ (Fig. 4*B*,*F*,*J*). For all the fits obtained on the numerical simulation curves, we obtained an error estimation R-square ≥ 0.97. Time constants τ of experimental and simulated astroglial membrane potentials were calculated by computing the rise and decay times between 20% and 80% of the maximal peak amplitude responses (Fig. 2*D*,*H*,*L*).

All time constants τ were fitted to curves using a single exponential$\left(e^{-\frac{t}{\tau }}\right)$. For all the fits obtained on the numerical simulation curves, we obtained an error estimation R-square ≥ 0.97.

**Approximation of facilitation/depression model parameters**. To account for the synaptic properties of CA1 pyramidal neurons following single, tetanic and repetitive stimulations, we generated a synaptic current using the depression-facilitation model (equation 1) where *I*_*app*_ depends on the input functions *f*_*s*_(*t*) (equation 4), *f*_*TT*_(*t*) (equation 5) and *f*_*Rs*_(*t*) (equation 6), respectively (Fig. 2*A*,*E*,*I*). The synaptic current parameters were fitted to experimental recordings [26] by matching the time of maximal peak amplitude of fEPSP with the one of *I*_*app*_ in control conditions (*τ* = 300 *ms*, *τ*_*inact*_ = 200 *ms*). The parameters for the K_ir_4.1 inhibition condition in the model were extracted from our experimental results on K_ir_4.1 glial conditional knockout mice [26] and are given by *τ*_*rec*_ = 500 *ms*, *τ*_*inact*_ = 160 *ms*. When K_ir_4.1 channels are inhibited (model) or knocked-out (experiment), the maximal peak amplitudes of the applied synaptic currents in the model (I_*app*_) and fEPSPs recorded experimentally are increased compared to control conditions [26].

### Simulation of neuronal firing at various frequencies

We imposed an initial input at various frequencies (0.1, 1, 3, 5, 10, 50 Hz). Each input is generated by a sub-firing square current lasting 5 ms (I_*app*_). In addition, we added a Brownian noise of amplitude σ = 0.68 *pA* ^2^ *ms* ^-1^ to induce neuronal membrane potential fluctuation (equation 21), which amplitude (1 mV) was chosen to induce a probabilistic firing of 0.2, matching the CA1 pyramidal cells synaptic release probability p = 0.2 (probability to induce a postsynaptic response in equation 1) [70]. Using the tri-compartment model, we simulated at various frequencies a quantity that we called the observed firing probability defined empirically at time *t* as the time dependent ratio of the number of spikes observed at time *t* to the total number of simulations.

## Supporting Information

S1 Fig Neuronal firing and extracellular K^+^ transients evoked by neuronal stimulations. Simulated neuronal firing (***A*,*C*,*E***) and [K^+^]_o_ (***B*,*D*,*F***) in control conditions (blue) following single (***A*,*B***), tetanic (100 Hz, 1 s) (***C*,*D***) and repetitive (10 Hz, 30 s) (***E*,*F***) stimulations.
(EPS)

S2 Fig Astroglial net potassium uptake changes with extracellular K^+^ levels. ***A-C***) Astroglial net K^+^ uptake (equation 27) and activity-dependent changes in [K^+^]_o_ evoked by single (light blue), tetanic (100 Hz, 1s, green) and repetitive (10 Hz, 30 s, dark blue) stimulations are plotted as a function of time. ***D***) Phase diagram illustrating astroglial K^+^ uptake as a dynamic function of activity-dependent changes in [K^+^]_o_ evoked by the different stimulations. Astroglial K^+^ net uptake is normalized to the maximum value obtained during repetitive stimulation.
(EPS)
